# Impacts of Internet Use on Chinese Patients’ Trust-Related Primary Healthcare Utilization

**DOI:** 10.3390/healthcare10102114

**Published:** 2022-10-21

**Authors:** Jiao Lu, Jingyan Bai, Qingqing Guo, Zhongliang Zhou, Xiaowei Yang, Qi Yu

**Affiliations:** 1 School of Public Policy and Administration, Xi’an Jiaotong University, Xi’an 710049, China; 2 School of Public Health, Shanxi Medical University, Taiyuan 030001, China; 3 School of Management, Shanxi Medical University, Taiyuan 030001, China

**Keywords:** primary healthcare, internet use, marginal effect, patients’ trust

## Abstract

Background: The internet has greatly improved the availability of medical knowledge and may be an important avenue to improve patients’ trust in physicians and promote primary healthcare seeking by reducing information asymmetry. However, very few studies have addressed the interactive impacts of both patients’ internet use and trust on primary healthcare-seeking decisions. Objective: To explore the impact of internet use on the relationship between patients’ trust in physicians and primary healthcare seeking among Chinese adults 18 years of age and older to understand the varieties of effects in different cities. Methods: Generalized linear mixed models were applied to investigate the interactive impacts of internet use and patients’ trust in physicians on primary healthcare seeking using pooled data from the China Family Panel Study of 2014 to 2018. We also compared these effects based on different levels of urbanization, ages, and PHC services. Results: Overall, a higher degree of patients’ trust (*p* < 0.001) was able to directly predict better primary healthcare seeking, and internet use significantly increased the positive effect of patients’ trust on primary healthcare seeking (*p* < 0.001). However, the marginal effect analysis showed that this effect was related to the level of patients’ trust and that internet use could reduce the positive effect of patients’ trust on primary healthcare seeking when the individual had a low level of trust (≤ 3 units). Further, the heterogeneity analysis indicated that the benefits from internet use were higher in cities with high urbanization, high aging, and high PHC service levels compared to cities with low levels of these factors. **Conclusions:** The internet use may enhance patients’ trust-related PHC utilization. However, this impact is effective only if patients’ benchmark trust remains at a relatively high level. Comparatively, the role of internet use is more effective in areas with high urbanization, high aging and high PHC level. Thus, with increasing accessibility to the internet, the internet should be regulated to disseminate correct healthcare information. Moreover, in-depth integration of the internet and PHC should be promoted to provide excellent opportunities for patient participation, and different strategies should be set according to each city’s characteristics.

## 1. Introduction

Primary healthcare (PHC) is the core element of any healthcare system due to its key role in balancing the two goals of equity and efficiency in the health service system [1]. It can not only alleviate the undersupply of regional healthcare services caused by the imbalanced allocation of health resources and improve the overall efficiency of health systems, but also help patients achieve better health outcomes at relatively low costs [2,3]. However, the introduction of market mechanisms in the healthcare system after 1978 undermined the economic and social foundations of PHC. PHC was at a disadvantage in the competition with larger hospitals and became a symbol of low-quality service, which made the public lose trust in the physicians’ service of PHC. Furthermore, the residents’ distrust in physicians of PHC did not reverse even though the government has pushed a new wave of efforts in public health infrastructure improvement, professional investments and incentive measures to improve the service quality of PHC [4,5].

Patients’ trust in physicians can be defined as a collection of patients’ expectations from physicians [6,7], thus reflecting each patient’s degree of reassurance or confidence in his or her physician [8]. This trust is the psychological mechanism used by patients to handle disease problems in healthcare contexts characterized by both vulnerability and uncertainty [9,10] and has a critical determinant role in patients’ healthcare-seeking decisions [11,12,13,14]. Previous studies found that patients’ low trust may restrict their utilization of PHC in China, which leads them often bypass primary care and access the health system at higher-level hospitals when they require medical treatment, even for mild health conditions [15]. A patient’s trust in a physician includes both the patient’s trust in the physician’s medical ability (cognition-based trust) and emotional dependence (affect-based trust) [16,17]. The information asymmetry between patients and physicians firstly damaged patients’ cognition-based trust in physicians, which further reduces physician–patient interactions and leads to a decline in patients’ affect-based trust in physicians [18]. These both lead to the decrease in patients’ trust in physicians.

Fortunately, the rapid development of mobile health, which is defined as the use of mobile device communication technology to connect with the internet anytime and anywhere in modern healthcare solutions to provide medical related services [19]. Searching the internet has become an increasingly popular channel for the public to seek health information. Thus, internet use may become an effective way to increase patients’ trust in a physician because that health information on the internet can serve as a supplement to reduce information asymmetry between patients and physicians [20,21]. Some researchers noted that online health communities provide a platform where patients can obtain health information and emotional support from other patients or physicians, help foster positive interactions between patients and physicians, and improve patients’ trust in their physicians [18]. However, other researchers argued that patients seeking information online may have adverse effects on their relationships with physicians [22,23]. Patients may be suspicious of medical professionals when the medical information obtained online is inconsistent with the physician’s advice [24]. Therefore, the effects of ubiquitous internet use on patients’ trust and PHC utilization represent an urgent problem to be solved.

Moreover, trust constitutes systematic vulnerability [25,26] and has a dynamic nature [27,28], as the pursuit of trust requires awareness of one’s confidence that the trusted party will do no harm, while it can be easily disrupted by even small day-to-day encounters. Thus, trust must be evaluated on an ongoing basis [29], as trust has a trajectory—not a static nature—that can change positively or negatively, multiple times [24]. These characteristics of trust should be of more concern in the era of the internet where information is ubiquitous and easily accessible [30]. However, current studies still lack evaluations of the dynamic effects of patient trust on PHC utilization, and the influence of internet use on these effects; such evaluations remain necessary to identify these complex interactions. Therefore, the purposes of the present study were (i) to examine the effect of patients’ trust in physicians on PHC utilization, (ii) to analyze whether internet use could increase patients’ trust in physicians and guide patients to seek healthcare from PHC providers, and (iii) to understand the variations of these effects in different cities. Additionally, corresponding to the study purposes, the research hypotheses in our study were (i) the patients’ trust in physicians may increase their utilization of PHC service, (ii) internet use may have a positive moderating effect on the relationship of patients’ trust in physicians and their primary health service utilization, and (iii) the interactive relationship among internet use, patients’ trust in physicians and their primary health service utilization may be varied with the different levels of urbanization, ages, and PHC services. Compared to current cross-sectional studies, this longitudinal study may make two contributions to current literature. First, a more robust examination of prospective relationships among patients’ trust in physicians, internet use and patients’ PHC utilization, enabling firmer conclusions to be drawn. Second, a more sophisticated analysis of a moderator of internet use on the relationship between patients’ trust in physicians and PHC utilization among different cities, helping the differentiation promotion strategies of patients’ PHC utilization to be clarified.

The paper architecture is arranged as follows: the first section introduces the literature review, theoretical mechanisms, and the aims and contributions of the paper. The second section describes materials and methods, including data collection, variable definitions, statistical analysis, and methodology. The third part displayed the results of empirical analysis, including the baseline regression, the heterogeneity analyses, and robustness tests. The fourth part discusses the valuable results. The last section describes the conclusion and policy implications.

## 2. Materials and Methods

### 2.1. Data Collection

Data were obtained from the China Family Panel Studies (CFPS) (This study was carried out by the ISSS of Peking University and surveyed approximately 15,000 households nationwide, using a multistage probability proportional-to-size sampling method. The researchers interviewed all members of the family in each sampled household, covering 621 villages/communities from 25 of China’s 30 provinces. The population of the sample collection area accounted for 94.5% of the country’s total population), which is a nationally representative, annual, large-scale longitudinal micro-integrated social survey study supervised by the Institute of Social Science Survey (ISSS) of Peking University, covering five rounds of data collection in 2010, 2012, 2014, 2016, and 2018 [31]. The questionnaire gathered individual-, family-, and community-level information on demographic and socioeconomic variables, as well as information on the respondents’ health.

Our study primarily used data from 2014, 2016, and 2018 since the CFPS not only involved an extensive set of measures on internet access and usage but also used the same set of test questionnaires to measure the health outcomes and sociodemographic characteristics needed for our study. The present study focuses on adults aged 18 years or older. The original sample from CFPS 2014 included 34,147 respondents in 192 counties (districts) and 25 provinces. CFPS followed up with 36,892 and 32,669 respondents in 2016 and 2018. We used the RStudio 1.1.456 software (RStudio, Inc.) to collate and clean the data and applied a random forest algorithm to fill in the missing values of potential confounders (< 10%) after removing duplicate records and missing samples of core variables. The final sample included 31,422, 33,161, and 29,989 respondents in 2014, 2016, and 2018, respectively.

#### Variables

The dependent variable in this study is PHC-seeking. Referring to the measures of Zhou [32], a question asking “where do you usually go to seek health services when you are sick?” (Table 1) was administered to assess participants’ PHC-seeking activities; we set this measure as a binary variable according to the level of hospital use [33]. The choice of primary care facilities, including community health centers/township health centers and community health service stations/village clinics, was scored as 1 or 0 (for none of the above).

The independent variables were the patients’ trust in local physicians and internet use. The question of “How much do you trust the local physicians?” was used to measure the trust degree in physicians among individuals and ranged from 0 (very high distrust) to 10 (very high trust), where a higher score indicated greater trust in local physicians. Internet behavior was determined by asking “How long do you participate in surfing the internet each week (≤ 70 h)?” on the questionnaire. The longer an individual participated in surfing the internet, the more likely he/she was to search for healthcare information on the internet [34,35].

In light of the existing literature [36,37,38,39,40,41], we controlled the socioeconomic characteristics and health information variables that could influence healthcare-seeking decisions. The baseline characteristics of respondents are shown in Table 1. Additionality, patients’ trust in local physicians, internet access, and healthcare-seeking decisions were also closely related to the public service capacity and healthcare service demands of the cities in which they lived. Thus, we further analyzed city disparities (population aging, urbanization, and PHC levels) in the effect of internet use on patients’ trust and PHC seeking. The levels of population aging, urbanization, and PHC service were evaluated based on data from the “China Statistical Yearbook (2015, 2017, and 2019)” and “China Health Statistics Yearbook (2015, 2017, and 2019)”. Population aging was based on the proportion of the total population aged 60 and over in the city. The urbanization level was calculated by dividing the urban population with the total population (including agricultural and non-agricultural residents). The PHC service level was evaluated by integrating the index by the TOPSIS method [42,43], including the number of people covered by each PHC institution and other six indicators [44]. When the corresponding value of the city was less than the median value of the total city, it was defined as a low-level urbanization/aging/primary healthcare area; otherwise, it was divided into a high-level urbanization/aging/primary healthcare area.

### 2.2. Statistical Analysis and Methodology

The mean and standard deviation for quantitative data and the rate or composition ratio for qualitative data were described for Chinese adults aged ≥ 18 years old (Table 1). Then, because longitudinal data have a potential lack of independence, a mixed-effects binomial logit model was established using GLIMMIX Proc Step in the SAS software, version 9.4 (SAS Institute Inc.), to test our hypotheses. Generalized linear mixed models (GLMMs) are based on generalized linear models that introduce random effects into the mean equation to deal with the correlation between repeated measurements and can accommodate clustered or over-dispersed data [45]. All tests were two-sided at a significance level of α = 0.05, and *p* < 0.05 indicated statistical significance.

**Figure 1:**
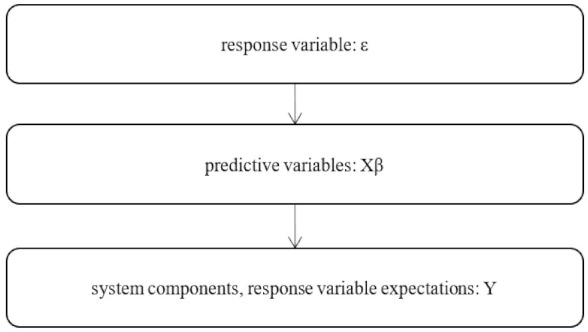
Generalized linear mixed flow chart of the model.

Mixed effect logistic regression is used to model binary outcome variables, where the logarithmic probability of results when data are grouped or when there are fixed and random effects is modeled as a linear combination of predictive variables (Figure 1).

The basic model is

$$Y_{\mathrm{ij}}=\gamma_{0}+\gamma_{1}X+\left(u_{\mathrm{oj}}+e_{\mathrm{ij}}\right)$$

among, $u_{\mathrm{oj}}$: random effect affecting the intercept; $e_{\mathrm{ij}}$:fixed effect

In addition, generalized linear mixed models were applied to investigate the interactive impacts of internet use and patients’ trust in physicians on primary healthcare seeking. It is a model construction of the integration of general linear and generalized linear models to transform the results of linear prediction to the response variables.

The basic model is

$$Y=X_{\beta }+Z_{u}+\varepsilon$$

compositive for four parts: Y: link function; $X_{\beta }$: fixed effects; $Z_{u}$: random effects; ε: error structure.

## 3. Results

**Figure 2:**
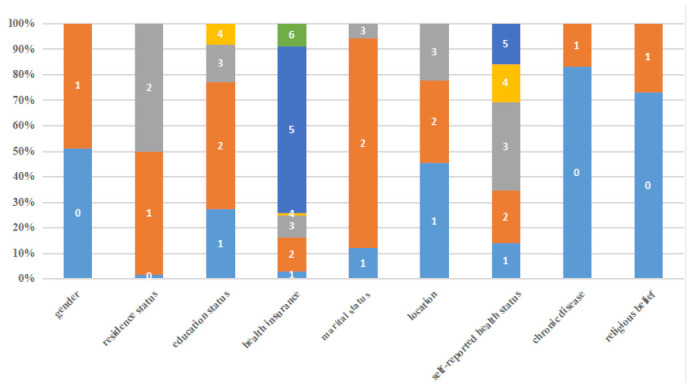
Descriptive of control variable. Note: Gender (0 = female; 1= male), residence status (0 = miss; 1 = urban; 2 = rural), education status (1 = illiterate; 2 = primary school or middle school; 3 = high school/secondary school/technical school/junior college; 4 = university or above), health insurance (1 = public medical care; 2 = urban employee medical insurance; 3 = urban resident medical insurance (including one old and one small insurance); 4 = supplementary medical insurance; 5 = new rural cooperative medical insurance; 6 = none of the above urban employees basic medical insurance), marital status (1 = unmarried; 2 = married/living together; 3 = divorced/widowed), location (1 = east; 2 = central; 3 = west), self-reported health status (1 = excellent; 2 = very good; 3 = good; 4 = fair; 5 = poor), diagnosis with a chronic disease, such as hypertension, diabetes, coronary heart disease, or stroke (0 = without chronic disease; 1 = with chronic disease) and whether they have religious belief (0 = no; 1 = yes).

In this section, we first describe the research results of control variables, which are shown in Figure 2. Second, we present the baseline model to explore the effect of patients’ trust in physicians and internet use on PHC seeking. Third, we estimate the models for different cities depending on the urbanization rate, aging rate, and PHC level. Last, we assess the robustness of the results by following the instrumental variable approach and replacing the model.

### 3.1. The Impact of Patients’ Trust in Physicians and Internet Use

Table 2 presents the interactive effects of patients’ trust in physicians and internet use on PHC seeking. Model 1 and Model 2, respectively, report the main effects of patients’ trust and internet use on PHC seeking. The results indicated that patient trust has a significantly positive relationship with primary healthcare seeking (β = 0.055, *p* < 0.01). Conversely, internet use has a negative effect on primary healthcare seeking (β = −0.002, *p* < 0.01). However, the positive and significant interactive effect of patients’ trust in physicians and internet use on primary healthcare seeking (β = 0.0008, *p* < 0.01) revealed that internet use has significantly enhanced the positive effects of patients’ trust in physicians on primary healthcare seeking. However, this result appeared paradoxical considering the negative effect of internet use on primary healthcare seeking (β = −0.002, *p* < 0.01). Thus, we further analyzed the marginal effect of internet use on the relationship between patients’ trust in physicians and primary healthcare seeking. The results showed that, overall, the marginal effect of internet use on primary healthcare seeking increased and was statistically significant with an increase in the degree of patients’ trust in physicians (*p* < 0.01). When the interactive effect of patients’ benchmark trust in physicians on primary healthcare seeking was greater than three units along with internet use, the predicted marginal value for seeking PHC was higher (*p* < 0.01); while the opposite effect was observed when the effect of patients’ benchmark trust in physicians was less than three units (*p* < 0.01) (Table 3). This result demonstrated that the effect of internet use on the relationship between patients’ trust in physicians and primary healthcare seeking is related to the degree of patients’ benchmark trust. When the individual has a low level of trust, internet use may decrease the positive effect of that individual’s trust in physicians on primary healthcare seeking; however, when the individual has a high level of trust, internet use tends to increase the positive effect of patients’ trust in primary healthcare seeking.

(1) Other variables defined in Table 1 are also controlled, but not reported, in all specifications, * is interactive effect of patients’ trust in physicians and internet use on primary healthcare-seeking.
(2) *** *p* < 0.01; ** *p* < 0.05; * *p* < 0.10.

**Table 3:** Estimation of the marginal effect on primary healthcare seeking.

Primary Healthcare Seeking		dy/dx		Delta-Method Std. Err.	t	*p*
Internet use		0.570				
Patients’ trust in physicians		0.0009				
Patients’ trust in physicians		1	−0.002 (−0.003~−0.002)	0.0002	−14.86	0
		2	−0.001 (−0.002~−0.0010)	0.0002	−7.5	0
		3	−0.0004 (−0.0008~0.00002)	0.0002	−1.84	0.04
		4	0.0006 (0.00007~0.001)	0.0002	2.25	0.024
		5	0.001 (0.0009~0.001)	0.0003	5.22	0
		6	0.002 (0.002~0.003)	0.0003	7.41	0
		7	0.003 (0.003~0.004)	0.0004	9.07	0
		8	0.004 (0.004~0.005)	0.0004	10.37	0
		9	0.005 (0.004~0.006)	0.0005	11.4	0
		10	0.006 (0.005~0.007)	0.0005	12.24	0

### 3.2. Effects of Urbanization, Aging, and Level of Primary Healthcare Service on Primary Healthcare Seeking

Due to its policy of supporting the rich first, China has a great disparity in its regional economic development [46]. This disparity led to the migration of the population and the aggregation of resources from low-level to high-level areas of economic development. The levels of regional urbanization, aging, and the PHC service are inevitable influencing factors of primary healthcare seeking among patients. Thus, we conducted a heterogeneity analysis to study the mechanism of the impact of internet use on primary healthcare seeking due to patients’ trust in physicians and marginal effect, based on urbanization, aging, and the PHC levels of different cities. The analysis results were consistent with those of the baseline model, even for different cities with different levels of urbanization, aging and PHC (Table 4 and Supplementary Table S1). Whatever the cities whit any levels of urbanization, age and PHC, the patients’ trust in physicians has a positive effect on primary healthcare seeking, internet use has a negative effect on primary healthcare seeking, and the interactive effect of patients’ trust in physicians and internet use has a positive effect on primary healthcare seeking. Comparatively, the effect of internet use on the relationship between patients’ trust in physicians and primary healthcare seeking in cities with high levels of urbanization, aging, and PHC was greater than that of low-level cities (Table 5).

### 3.3. Robustness Checks

Because trust is intertwined with perceived service quality and thus affects primary healthcare seeking [13,47], the causality between patients’ trust in physicians and primary healthcare seeking can be bidirectional and circular [48]. Meanwhile, in order to avoid the potential issue of omitted variables and measurement errors, we selected trust in local government officials as an instrumental variable to test the robustness of the original interactive model. We chose this variable because government action can greatly affect the public’s trust in public employees depending on the social policies being proposed [49]. Individuals’ trust in the government was significantly related to trust in physicians. Moreover, the degree of individuals’ trust in local government officials did not affect the random disturbance item, indicating its exogenous nature. Based on the results, the interactive effect of individuals’ trust in physicians and internet use on primary healthcare seeking remained robust after the introduction of instrumental variables (Supplementary Table S2).

For comparison, the generalized estimating equation (GEE) was used to further estimate the model. It was found that the regression results of all models were consistent with those of the baseline regression results—that is, internet behavior can enhance the positive impacts of patients’ trust in physicians on an individual’s primary healthcare seeking (*p* < 0.01). Conversely, internet use has a negative effect on primary healthcare seeking (β = −0.019, *p* < 0.01). Additionally, there is a positive and significant interactive effect of patients’ trust in physicians and internet use on primary healthcare seeking (β = 0.005, *p* < 0.01) (Table 6).

## 4. Discussion

To reverse the public’s under-utilization of PHC has always been a major concern in China, and our study verified that it is partly due to the lack of patients’ trust in local physicians, which is similar to previous studies [50,51]. According to social exchange theory, trust is typically associated with high-quality communication and benign interactions between patients and providers [52]; trust facilitates disclosure by the patient and gives the patient greater confidence in decision making regarding healthcare seeking [53,54]. In China, patients are more worried about the uncertainty and risk of the competence and intentions of PHC providers compared to secondary and tertiary medical service institutions. Trust may help improve patients’ perceptions of healthcare providers [54], then enhance their loyalty, further balance their risk and uncertainty perceptions of service delivery processes [10,13,55], and provide the basis for patient’s decisions to seek healthcare through PHC. However, the information asymmetry between patients and providers [56], and the fact that they have become increasingly empowered to make informed decisions [10,57], influenced the patients’ trust in physicians. Coincidentally, the increase in internet accessibility has enabled the public to seek information (e.g., to search for conditions, symptoms, and treatment options) online before or after visiting physicians [58,59], which is expected to alleviate the traditional information asymmetry between physicians and patients, leading to a profound impact on the trust of patients in their physicians and changing patients’ healthcare choice behaviors even further.

We found that patients’ trust in physicians is conditional and subject to an iterative process wherein patients test the trustworthiness of physicians against their expectations [60]. Although internet use positively moderated the promoting effect of patients’ trust in physicians on primary healthcare seeking, the marginal effect revealed that internet use was found to be detrimental to patients seeking healthcare from PHC institutions when they have very low levels of benchmark trust in local physicians. Internet health information seeking is an active effort, in which an individual searches for information to satisfy a health-based informational need or goal [18]. Information processing theory states that people driven by high cognition will ensure their physicians are reliable by searching and collecting supplementary information to assess the information provided by physicians. For patients who have high-level trust in physicians, the positive attitude of Internet health information seeking also means that such patients are more likely to actively communicate with service providers about information obtained online, which is likely to ameliorate patients’ fear of service providers’ opportunism [7] and further solidify patient trust. By contrast, the distrust of patients with low-level benchmark trust in physicians can easily lead them to the acquisition of inaccurate, misleading, or confusing information. This information can negatively influence patients’ attitudes, beliefs, and opinions in physicians [32], then hindering their access to PHC [61]. Moreover, good communication between patients and physicians will achieve patients’ personalized health counseling needs to increase their cognition-based trust in physicians, also promoting feelings of psychological safety among patients with physicians to increase their affect-based trust in physicians [58,62], thereby improving their adherence to PHC.

Furthermore, we found that compared to their respective counterparts, the positive effect of patients’ trust on PHC utilization was stronger in areas with high urbanization, high aging, and low PHC levels, while the moderated effect of internet use was higher in high-urbanization and high-aging areas, as well as in areas with high PHC levels. The urban–rural dichotomy in China has also created an urban–rural disparity in PHC. The population flow from rural areas due to urbanization experiences higher quality healthcare services, thereby increasing patients’ trust in local physicians and the effect of patients’ trust in physicians on PHC utilization [63]. Urbanization also increased internet accessibility among these populations and strengthened the effect of internet use. Those among the Chinese aging population not only tend to pay more attention to their own health and frequently communicate with PHC providers but also prefer to gather together to spread online health information and enhance the effect of patients’ trust on primary healthcare seeking [64,65]. Importantly, there are few options for patients in low-level PHC areas [66]; thus, trust in physicians may become an important factor that affects the decisions of this group. Conversely, patients in high-level PHC areas are more likely to have good interactions with their physicians, so internet use may help increase the effect of patient trust in physicians on PHC utilization in such areas [67].

There are several limitations to this study. First, although this study employed nationally representative large-scale micro-integrated social-survey longitudinal data to analyze the relationships between individuals’ trust in physicians, internet use, and PHC-seeking behavior, the self-reported data from the face-to-face interviews may have introduced an acquiescence bias, social desirability bias, or interviewer bias. Second, due to data limitations, when interpreting the results, only the internet behavior of “online browsing duration” was considered, rather than also including interactive internet medical behaviors such as online consultations with physicians and healthcare information seeking, which would more thoroughly present the impact of the internet on residents’ healthcare-seeking behaviors. Third, even though the study findings were positive after controlling for multiple confounding factors, the survey was conducted several years ago. Thus, some results might not fully capture recent reform changes. This factor requires further evaluations over longer time intervals. Future studies may need more robust designs with better external validity to verify these conclusions.

## 5. Conclusions

Theoretically and empirically, internet use is an important factor that fosters patient engagement in medical decisions and makes patients aware of “what to do”, rather than just being told “how to do it” by physicians [23]. However, this ‘mutual participation’ may lead to contradictory results depending on the patients’ benchmark trust in local physicians. Meanwhile, the role of internet information seeking was found to be more effective in areas with high urbanization, high aging, and high PHC levels. Therefore, to avoid some cities falling into a vicious circle of PHC utilization with the increasing accessibility of the internet, the government should enhance patients’ benchmark trust in local physicians and disseminate correct healthcare information on the internet. Healthcare professionals should view the internet as an educational tool and construct a transparent medical information mechanism to provide greater opportunities for patient participation; policy makers should set differentiation strategies according to each city’s characteristics, continually improve the service capacity of cities with low-level PHC service by improving the quality of infrastructure and human capital, and develop digital medical equipment and diagnosis and treatment APPs that are suitable for older adults.

## Figures and Tables

**Table 1:** Variable definitions and summary statistics.

	Description	Observation (N = 28,721)	Description (%/X ± SD)
Dependent variables			
The primary healthcare seeking	1 = Primary care facilities	18,292	63.7
	0 = Otherwise	10,429	36.3
Independent variables			
Patients’ trust in physicians	0~10	28,721	6.81 ± 2.345
Internet use	Hours	28,721	3.197 ± 8.124
Control variables			
Gender	1 = Male	14,048	48.9
	0 = Female	14,673	51.1
Age (years)			
Residence status	0 = Miss	475	1.7
	1 = Urban	13,887	48.4
	2 = Rural	14,359	50.0
Education status	1 = Illiterate	7850	27.3
	2 = Primary school or Middle school	14,275	49.7
	3 = High school/secondary school/technical school/Junior college	4185	14.6
	4 = University or above	2411	8.4
Health insurance	1 = Public medical care	855	3.0
	2 = Urban employee medical insurance	3813	13.3
	3 = Urban resident medical insurance (including one old and one small insurance)	2486	8.7
	4 = Supplementary medical insurance	209	0.7
	5 = New rural cooperative medical insurance	18,790	65.4
	6 = None of the above	2568	8.9
Marital status	1 = Unmarried	3470	12.1
	2 = Married/living together	23,605	82.2
	3 = Divorced/Widowed	1646	5.7
Location	1 = East	13,023	45.3
	2 = Central	9289	32.3
	3 = West	6409	22.3
Self-reported health status	1= Excellent	4050	14.1
	2 = Very good	5853	20.4
	3 = Good	9975	34.7
	4 = Fair	4296	15.0
	5 = Poor	4542	15.8
Chronic disease	1 = Yes	4874	17.0
	0 = No	23,847	83.0
Religious belief	1 = Yes	7776	27.1
	0 = No	20,945	72.9
Heterogeneity analysis variables			
Aging level	The total population aged 60 and over/total population		
Urbanization level	The urban population/total population		
PHC service level	The number of people covered by each PHC institution (total urban population/number of PHC institutions) The number of PHC personnel per 10,000 people (number of PHC staff/10,000 people) The number of beds in PHC institutions per 10,000 people (number of beds in PHC institutions/10,000 people) The average number of diagnoses and treatments per year in each PHC institution (number of diagnoses and treatments/number of PHC institutions) The average number of hospital admissions per year in each PHC institution (number of hospital admissions/number of PHC institutions) The utilization rate of hospital beds in community health service The utilization rate of hospital beds in township–village health centers		

Data source: China Family Panel Studies (CFPS), 2014–2018. Data source: “China Statistical Yearbook”, “China Health Statistics Yearbook”, 2015, 2017 and 2019.

**Table 2:** Models for the interactive effects of patients’ trust in physicians and internet use on primary healthcare seeking.

Primary Healthcare Seeking	Model 1	Model 2	Model 3
Patients’ trust in physicians	0.055 (<0.01)		0.048 (<0.01)
Internet use		−0.002 (<0.01)	−0.0007 (<0.01)
Patients’ trust in physicians * Internet use			0.0008 (<0.01)

**Table 4:** Estimation of the marginal effect on primary healthcare seeking for different cities.

		Patients’ Trust in Physicians	Internet Use	Patients’ Trust in Physicians * Internet Use
Primary healthcare seeking				
Low-level urbanization	Model 1	0.049 (<0.01)		
	Model 2		−0.002 (<0.01)	
	Model 3	0.043 (<0.01)	−0.001 (<0.01)	0.007 (<0.01)
High-level urbanization	Model 1	0.062 (<0.01)		
	Model 2		−0.002 (<0.01)	
	Model 3	0.054 (<0.01)	−0.004 (<0.01)	0.009 (<0.01)
Low-level aging	Model 1	0.053 (<0.01)		
	Model 2		−0.002 (<0.01)	
	Model 3	0.048 (<0.01)	−0.008 (<0.01)	0.007 (<0.01)
High-level aging	Model 1	0.056 (<0.01)		
	Model 2		−0.002 (<0.01)	
	Model 3	0.049 (<0.01)	−0.006 (<0.01)	0.001 (<0.01)
Low-level primary healthcare	Model 1	0.056 (<0.01)		
	Model 2		−0.002 (<0.01)	
	Model 3	0.050 (<0.01)	−0.008 (<0.01)	0.007 (<0.01)
High-level of primary healthcare	Model 1	0.053 (<0.01)		
	Model 2		−0.002 (<0.01)	
	Model 3	0.046 (<0.01)	−0.009 (<0.01)	0.001 (<0.01)

* is interactive effect of patients’ trust in physicians and internet use on primary healthcare-seeking.

**Table 5:** Estimation of the marginal effect on primary healthcare seeking of different level cities.

Primary Healthcare Seeking	Marginal Effects	Urbanization Level		Primary Healthcare Service Level		Aging Level	
		Low-Level	High-Level	Low-Level	High-Level	Low-Level	High-Level
Trust	Patients’ trust in physicians	0.049	0.064	0.0586	0.0540	0.0551	0.0574
	Internet use	0.0008	0.001	0.0009	0.0010	0.0007	0.0010

**Table 6:** Estimation results of the generalized estimating equation.

Primary Healthcare Seeking	Model 1	Model 2	Model 3
Patients’ trust in physicians	0.295 (< 0.05)		0.257 (0.003)
Internet use		−0.019 (<0.01)	−0.016 (<0.01)
Patients’ trust in physicians * Internet use			0.005 (<0.01)

* is interactive effect of patients’ trust in physicians and internet use on primary healthcare-seeking.

## Data Availability

The datasets generated and analyzed during the current study are available in the China Family Panel Studies repository, https://opendata.pku.edu.cn/dataverse/CFPS, accessed on 15 October 2021.

## Supplementary Materials

The following are available online at https://www.mdpi.com/article/10.3390/healthcare10102114/s1, Table S1: Estimation of the marginal effect on primary healthcare-seeking of different urbanization cities, aging, primary healthcare; Table S2: TOPSIS results.
