# Supplementary material for: Impacts of Internet Use on Chinese Patients’ Trust-Related Primary Healthcare Utilization
Source: Healthcare (Basel). 2022 Oct 21;10(10):2114. doi: 10.3390/healthcare10102114 (PMC9602738; doi:10.3390/healthcare10102114)
Supplement: Supplementary file 1 [file healthcare-10-02114-s001.zip › healthcare-1929590-supplementary.pdf]

Supplementary Table S1. Estimation of the marginal effect on primary healthcare-seeking of different urbanization cities, aging, primary healthcare

Hospital	dy/dx	Delta-method Std. Err.	t	P
low-level urbanization cities				
Internet time				
trust				
1	-0.0026(-0.0030~-0.0022)	0.0002	-12.65	0
2	-0.0017(-0.0022~-0.0013)	0.0002	-7.45	0
3	-0.0009(-0.0014~-0.0003)	0.0003	-3.20	0.001
4	-0.00004(-0.0007~0.0006)	0.0003	-0.10	0.923
5	0.00082(0.00006~0.0016)	0.0004	2.13	0.033
6	0.0017(0.0008~0.0025)	0.0004	3.75	0
7	0.0025(0.0015~0.0035)	0.0005	4.96	0
8	0.0034(0.0022~0.0044)	0.0006	5.89	0
9	0.0042(0.0030~0.0055)	0.0006	6.62	0
10	0.0051(0.0037~0.0064)	0.0007	7.21	0
high levels of urbanization cities				
Internet time				
trust				
1	-0.0018(-0.0023~-0.0014)	0.0002	-7.84	0
2	-0.0008(-0.0013~-0.0003)	0.0003	-2.93	0.003
3	0.0002(-0.0004~0.0009)	0.0003	0.68	0.497
4	0.0012(0.0005~0.0020)	0.0004	3.28	0.001
5	0.0023(0.0014~0.0031)	0.0004	5.18	0
6	0.0033(0.0023~0.0043)	0.0005	6.60	0
7	0.0043(0.0032~0.0054)	0.0006	7.70	0
8	0.0053(0.0041~0.0066)	0.0006	8.56	0
9	0.0064(0.0050~0.0077)	0.0007	9.25	0
10	0.0074(0.0059~0.0089)	0.0008	9.82	0
low aging level				
Internet time				
trust				
1	-0.0024（-0.0028~-0.0019）	0.0002	-10.1	0
2	-0.0016（-0.0022~-0.0011）	0.0003	-5.95	0
3	-0.0009（-0.0015~-0.0002）	0.0003	-2.68	0.007
4	-0.0001（-0.0009~-0.0006）	0.0004	-0.31	0.758
5	0.0006（-0.0003~-0.0015）	0.0005	1.39	0.164
6	0.0014（0.0004~0.0024）	0.0005	2.64	0.008
7	0.0021(0.0010~0.0033)	0.0006	3.58	0
8	0.0029（0.0016~0.0042）	0.0007	4.3	0
9	0.0036（0.0022~0.0051）	0.0007	4.88	0
10	0.0044（0.0028~0.0060）	0.0008	5.34	0

high aging level				
Internet time				
trust				
1	-0.0022（-0.0026~-0.0018）	0.0002	-10.82	0
2	-0.0011（-0.0016~-0.0007）	0.0002	-4.88	0
3	-0.0001（-0.0006~0.0004）	0.0003	-0.37	0.714
4	0.0009（0.0003~0.0016）	0.0003	2.91	0.004
5	0.0020（0.0012~0.0027）	0.0004	5.29	0
6	0.0030（0.0022~0.0038）	0.0004	7.07	0
7	0.0040（0.0031~0.0050）	0.0005	8.42	0
8	0.0051（0.0040~0.0061）	0.0005	9.48	0
9	0.0061（0.0049~0.0073）	0.0006	10.33	0
10	0.0071（0.0059~0.0084）	0.0006	11.02	0
low-level medical service areas				
Internet time				
trust				
1	-0.0021（-0.0026~-0.0016）	0.0003	-7.92	0
2	-0.0012（-0.0018~-0.0006）	0.0003	-4.03	0
3	-0.0004（-0.0011~0.0003）	0.0004	-1.05	0.296
4	0.0005（-0.0003~0.0013）	0.0004	1.15	0.251
5	0.0013（0.0004~0.0023）	0.0005	2.77	0.006
6	0.0022（0.0011~0.0033）	0.0006	3.98	0
7	0.0031（0.0018~0.0043）	0.0006	4.92	0
8	0.0039（0.0026~0.0053）	0.0007	5.66	0
9	0.0048（0.0033~0.0063）	0.0008	6.25	0
10	0.0056（0.0040~0.0073）	0.0008	6.73	0
high-levels medical service areas				
Internet time				
trust				
1	-0.0028（-0.0032~-0.0024）	0.0002	-14.08	0
2	-0.0018（-0.0023~-0.0014）	0.0002	-7.86	0
3	-0.0008（-0.0014~-0.0003）	0.0003	-3.04	0.002
4	0.0001（-0.0005~0.0008）	0.0003	0.42	0.676
5	0.0011（0.0004~0.0019）	0.0004	2.89	0.004
6	0.0021（0.0012~0.0030）	0.0004	4.71	0
7	0.0031（0.0021~0.0041）	0.0005	6.08	0
8	0.0041（0.0030~0.0052）	0.0006	7.13	0
9	0.0051（0.0038~0.0063）	0.0006	7.97	0
10	0.0061（0.0047~0.0074）	0.0007	8.65	0

The TOPSIS method consists of the following steps:

1. Determine the original matrix and normalize the decision matrix.

Collect the original data, determine the evaluation object and evaluation index, and get the original data matrix. And in general, there are benefit attributes and cost attributes in the MADM （Multiattribute Decision-making） problems. In order to measure all attributes in dimensionless units and facilitate inter-attribute comparisons, we introduce the following formulas to normalize each attribute value in decision original matrix into a corresponding element Aij.

$$A_{ij} = \frac{a_{ij}}{\sqrt{\sum_{i=1}^{n} a_{ij}^2}} \tag{1}$$

for benefit attribute a_{ij}, And

$$A_{ij} = 1 - \frac{a_{ij}}{\sqrt{\sum_{i=1}^{n} a_{ij}^2}} \tag{2}$$

for cost attribute a_{ij}.

2.Determine the positive and negative ideal solutions.

The PIS A+ and NIS A are determined, respectively, as follows:

$A^+ = (a_1^+, a_2^+, a_3^+ \cdots a_m^+)$,

$A^- = (a_1^-, a_2^-, a_3^- \cdots a_m^-)$,

among，

$$A_j^+ = \max_{1 \le i \le n} \{a_{ij}\} \tag{3}$$

$$A_j^- = \min_{1 \le i \le n} \{a_{ij}\} \tag{4}$$

j = 1, 2, 3,⋯,m

3.Measure the distance from positive and negative ideal solutions.

The separation of each alternative form the PIS, D_i^+ , is given as:

$$D_i^+ = \sqrt{\sum_j^m \left(a_{ij} - a_{ij}^+\right)^2} \tag{5}$$

Similarly, the separation form the NIS, D_i^- is given as:

$$D_i^- = \sqrt{\sum_j^m \left(a_{ij} - a_{ij}^-\right)^2} \tag{6}$$

4. Calculate the closeness coefficient to the ideal solutions.

The closeness coefficient of the ith alternative Ai with respect to the ideal solutions is defined as: $C_i = D_i^- / (D_i^+ + D_i^-)$

5.Rank the preference order.

A set of alternatives then can be ranked by preference according to the descending order of Ci; in other words, larger Ci means better alternative.

Supplementary Table S2. TOPSIS results

province	C_i	sequence
Shanghai	0.0661	1
Jiangsu	0.0512	2
Guangdong	0.0464	3
Shandong	0.0459	4
Henan	0.0441	5
Sichuan	0.0441	6
Hubei	0.042	7
Anhui	0.0419	8
Hunan	0.0379	9
Tianjin	0.0368	10
Yunnan	0.0361	11
Chongqing	0.0354	12
Guangxi	0.0346	13
Zhejiang	0.034	14
Beijing	0.0338	15
Hebei	0.0324	16
Hainan	0.0291	17
Guizhou	0.0285	18
Jiangxi	0.028	19
Xinjiang	0.0269	20
Heilongjiang	0.0268	21
Fujian	0.0252	22
Liaoning	0.024	23
Shanxi	0.0226	24
Gansu	0.0217	25
Shanxi	0.0201	26
Ningxia	0.0196	27
Qinghai	0.0193	28
Neimenggu	0.0186	29
Jilin	0.018	30
Xizang	0.0088	31
